# A case of skew deviation and downbeat Nystagmus induced by Lithium

**DOI:** 10.1186/s12886-019-1270-3

**Published:** 2019-12-16

**Authors:** Hyunkyu Hong, In Jeong Lyu

**Affiliations:** 1 Department of Ophthalmology, Nowon Eulji Medical Center, Eulji University School of Medicine, Seoul, Republic of Korea; 2 Department of Ophthalmology, Korea Cancer Center Hospital, Korea Institute of Radiological and Medical Sciences, #75 Nowon-ro, Nowon-gu, Seoul, Republic of Korea 01812

**Keywords:** Lithium, Nystagmus, Skew deviation

## Abstract

**Background:**

Lithium salts have been commonly used for prophylaxis and treatment of bipolar disorder and have numerous side effects. However, there has been no report of skew deviation and downbeat nystagmus associated with lithium. Herein, we report the first case of lithium-induced skew deviation and downbeat nystagmus.

**Case presentation:**

A 39 years-old woman presented with intermittent vertical diplopia and dizziness within 1–2 months. Ophthalmologic examination revealed downbeat nystagmus and 6 prism diopters of right hypertropia. Funduscopic examination showed mild incyclotorsion on right eye. However, ductions and versions were within normal range. Other neurological examinations were also normal. She had a history of bipolar disorder treated with daily 600-900 mg of lithium for past 6 years, and 2 months before the first visit, daily dose of lithium was increased to 1200 mg. We referred the patients to psychiatrist. Although the serum level of lithium was within the normal therapeutic range, her daily dose of lithium was reduced to 600 mg and then stopped. 6 days after cessation of lithium, down beat nystagmus and right hypertropia were completely resolved and symptoms did not recur over a year.

**Conclusion:**

Even within a normal therapeutic range, downbeat nystagmus and skew deviation can occur as side effect of lithium. Dehydration may contribute to the neurotoxicity of lithium.

## Background

Lithium salts are commonly used for prophylaxis and treatment of bipolar disorder. Lithium has numerous side effects, and they can occur even if serum lithium levels are within the normal therapeutic range [1]. Downbeat nystagmus can occur as a neurotoxic side effect of lithium use [2]. However, there has been no report of skew deviation and downbeat nystagmus associated with lithium. This is the first report of skew deviation in addition to downbeat nystagmus in a potential case of lithium toxicity.

### Case presentation

A 39-year-old woman visited the neuro-ophthalmologic clinic with intermittent binocular vertical diplopia, oscillopsia, and dizziness. She complained that these symptoms occurred 3–4 times over the past 1–2 months and resolved spontaneously within an hour. However, the symptoms persisted at presentation.

**Fig. 1:**
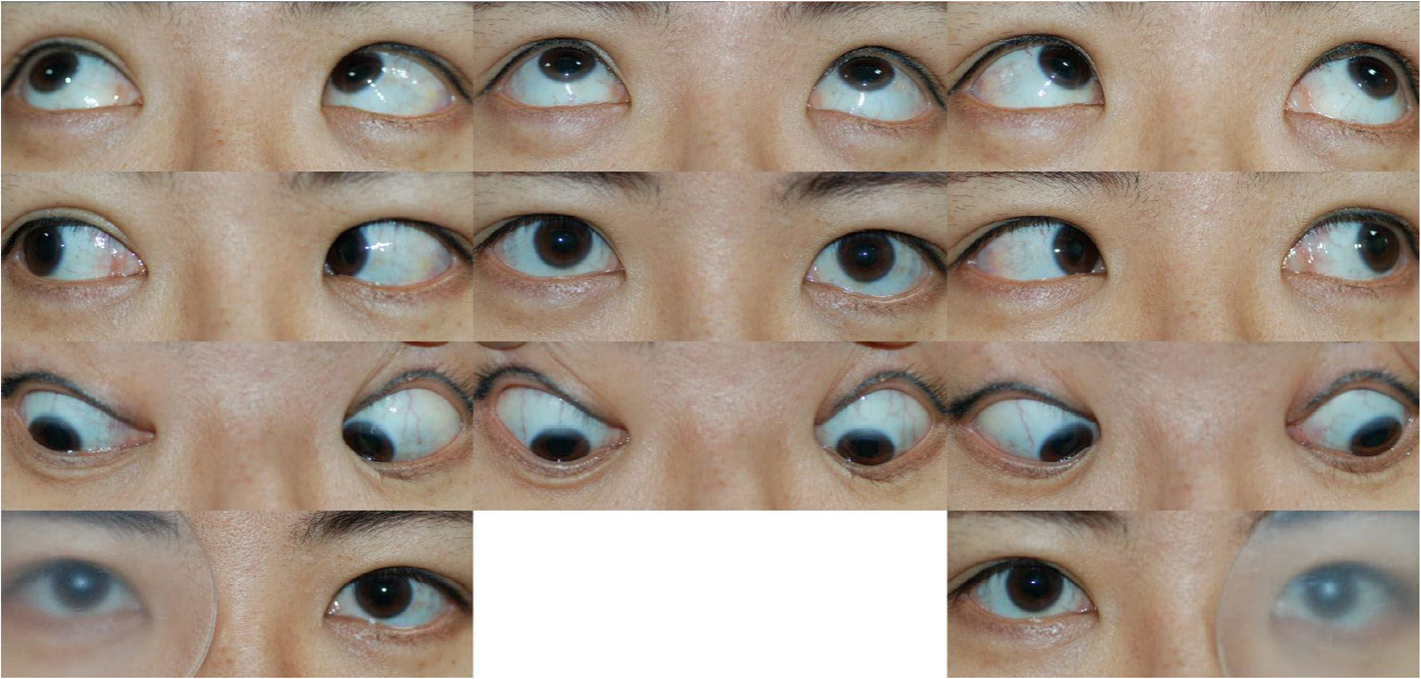
Nine-gaze photographs revealed normal ductions and versions. However, the patient demonstrated downbeat nystagmus and 6 prism diopters of right hypertropia on the alternative prism cover test

**Fig. 2:**
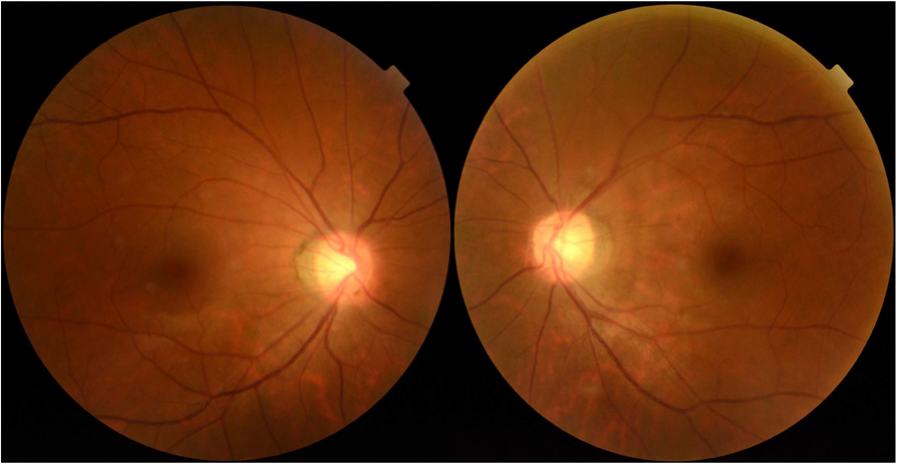
Fundus photographs demonstrated mild incyclotorsion in the right eye, and the left eye was normal

Her best-corrected visual acuity was 20/32 in the right eye and 20/40 in the left eye. Slit-lamp examination demonstrated cortical and posterior capsular lenticular opacities in both eyes. Although ductions and versions were normal, she demonstrated 6 prism diopters (PD) of right hypertropia and downbeat nystagmus (Fig. 1). Hypertropia was concomitant, even with the head tilt test. Downbeat nystagmus was observed in all gazes, but was slightly suppressed on upward gaze. On funduscopic examination, mild incyclotorsion was detected on her right eye (Fig. 2). All other neurological examinations revealed nonspecific findings.

She had a past medical history of bipolar disorder treated with lithium 600–900 mg per day for the past 6 years. Two months before her first visit, her daily dose of lithium was increased to 1200 mg to control her bipolar symptoms. Her other medications comprised quetiapine 175 mg/day, clonazepam 1 mg/day, valproate 1250 mg/day, lamotrigine 200 mg/day, propranolol 40 mg/day, topiramate 50 mg/day, and ziprasidone 40 mg/day. She had no history of alcohol abuse or relevant viral infection.

Interestingly, she experienced improvement in diplopia and oscillopsia just after consuming sports drinks or receiving intravenous fluid therapy. Therefore, we repeated the ophthalmic examination 20 min after she consumed sports drinks, and we confirmed that the nystagmus and hypertropia subsided after ingesting sports drinks.

Her evaluation plan included brain magnetic resonance image (MRI), laboratory work-up, and consultation with a psychiatrist to assess the possibility of lithium-induced side effects. Her serum lithium level was 0.8 mEq/L (therapeutic range: 0.6–1.0 mEq/L), and there was no evidence of electrolyte imbalance. The serum levels of sodium, potassium, calcium, and chloride were normal, and the vitamin B12 level was also normal.

After the psychiatric consultation, the lithium dosage was reduced 600 mg and then stopped within 1 week. However, the patient refused further exams, including brain MRI. Six days after cessation of lithium, downbeat nystagmus and right hypertropia completely resolved, and her symptoms did not recur over a period of 1 year. The patient provided written informed consent for publication.

### Discussion and conclusions

Downbeat nystagmus is a well-known potential presentation of lithium toxicity, while the most common ocular motor complication associated with lithium is horizontal gaze-evoked nystagmus [3]. Other ocular complications such as periodic alternating nystagmus, saccadic pursuit, oculogyric crisis, and opsoclonus have also been attributed to lithium toxicity [3].

Previous studies reported that the pathogenesis of lithium-induced downbeat nystagmus is damage in the nucleus prepositus hypoglossi (NPH), medial vestibular nuclei, and cerebellum [3, 4]. Although the exact mechanism of this damage remains unclear, it mostly results from chronic lithium exposure versus acute toxicity [3, 5]. Among those affected, lithium dosage varied from 500 to 1800 mg per day [3, 6, 7]. Downbeat nystagmus can occur at a therapeutic serum level of lithium and persist even after cessation of the drug [3, 6–10]. Factors associated with lithium toxicity included new medications altering renal function (such as angiotensin-converting enzyme inhibitors and nonsteroidal anti-inflammatory medications), dehydration, febrile infection, and gastroenteritis [11]. Although severe neurological symptoms can develop with accidental or intentional lithium overdoses, ocular manifestations by chronic toxicity may occur separately or with mild neurological symptoms like ataxia or tremor [8]. Blurred vision [6] and horizontal gaze palsy [3, 7] were reported as a neuro-ophthalmologic manifestation accompanying downbeat nystagmus.

Skew deviation is defined as vertical misalignment of the eyes due to asymmetric supranuclear input involving otolith-ocular pathways [12]. It can occur with a variety of abnormalities in the vestibular system, brainstem, or cerebellum and can be associated with other neurologic symptoms [13, 14]. Skew deviation and downbeat nystagmus in a patient with a lesion in the NPH, a key constituent of the vestibular-cerebellar-brainstem neural network, have been also reported previously [15]. However, to the best of our knowledge, this is the first report of skew deviation in addition to downbeat nystagmus in a potential case of lithium toxicity.

Another unique feature of our case is that the patient’s symptoms temporarily improved after intravenous fluid therapy or consuming a sports drink. Volume depletion regardless of the underlying origin is a common cause of chronic lithium intoxication and hydration helps maximize lithium clearance [11]. In this case, consumption of sports drinks and intravenous fluid therapy appeared to control the symptoms temporarily. Furthermore, our case emphasized that early recognition of neuro-ophthalmologic symptoms in cases suspicious of lithium toxicity despite a normal therapeutic level of lithium, and consequent prompt drug cessation, could increase the likelihood of avoiding irreversible sequelae [6, 8].

There are several limitations to this study. Brain MRI was not performed and serum magnesium level was not evaluated due to patient refusal. However, there was no history of alcohol abuse or relevant viral infection. The patient had normal neurological measures and normal serum levels of vitamin B12 and valproate, and electrolytes, reducing the likelihood of other causes of skew deviation and downbeat nystagmus. Other aetiologies of downbeat nystagmus include structural lesions of the brain and metabolic abnormalities, such as hypomagnesaemia, thiamine deficiency, and vitamin B12 deficiency [16, 17]. Lamotrigine toxicity has also been reported to be a rare cause of downbeat nystagmus, and its half-life is extended when used with valproate [18]. However, while our patient takes lamotrigine, she experienced vertical diplopia and nystagmus only after an increase in daily lithium dose, and symptoms resolved completely after cessation of lithium without other medication changes. This supports the presumption that both skew deviation and downbeat nystagmus can be attributed to lithium consumption in this case.

In conclusion, skew deviation and downbeat nystagmus can occur in patients with long-term lithium use, even within the therapeutic range, and symptoms may improve with cessation of the drug.

## Data Availability

The datasets supporting the conclusions of this article are included within the article.

## Abbreviations

MRI: Magnetic resonance imaging
NPH: Nucleus prepositus hypoglossi
PD: Prism diopters
